# Construction and Loss of Bacterial Flagellar Filaments

**DOI:** 10.3390/biom10111528

**Published:** 2020-11-09

**Authors:** Xiang-Yu Zhuang, Chien-Jung Lo

**Affiliations:** Department of Physics and Graduate Institute of Biophysics, National Central University, Taoyuan City 32001, Taiwan; xiangyu066@gmail.com

**Keywords:** self-assembly, injection-diffusion model, flagellar ejection

## Abstract

The bacterial flagellar filament is an extracellular tubular protein structure that acts as a propeller for bacterial swimming motility. It is connected to the membrane-anchored rotary bacterial flagellar motor through a short hook. The bacterial flagellar filament consists of approximately 20,000 flagellins and can be several micrometers long. In this article, we reviewed the experimental works and models of flagellar filament construction and the recent findings of flagellar filament ejection during the cell cycle. The length-dependent decay of flagellar filament growth data supports the injection-diffusion model. The decay of flagellar growth rate is due to reduced transportation of long-distance diffusion and jamming. However, the filament is not a permeant structure. Several bacterial species actively abandon their flagella under starvation. Flagellum is disassembled when the rod is broken, resulting in an ejection of the filament with a partial rod and hook. The inner membrane component is then diffused on the membrane before further breakdown. These new findings open a new field of bacterial macro-molecule assembly, disassembly, and signal transduction.

## 1. Introduction

**Figure 1:**
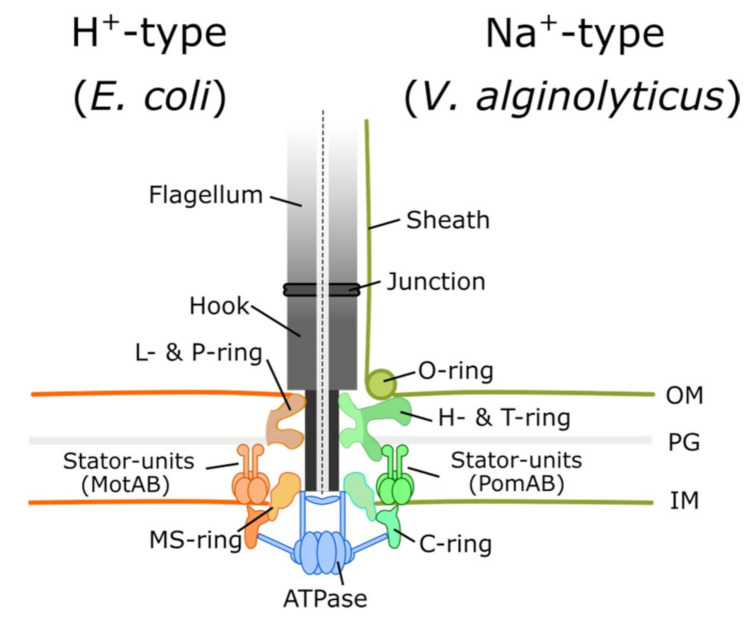
Bacterial flagellar motors are mainly classified into proton-driven and sodium-driven motors. Torque generation requires an interaction between stator units and FliG on the C-ring. In the proton-driven motor of *E. coli*, the stator is composed of MotA and MotB, whereas PomA and PomB are sodium-driven analogues in *V. alginolyticus*. The common elements for both motor types are LP, MS, and C rings. In *V. alginolyticus*, an additional sheath covers the flagellar filament.

Since Antonie van Leeuwenhoek observed animalcules by using his single-lens microscope in the 18th century, we have entered a new era of microbiology. The motility of single-cell organisms is fascinating, but it took a long period of time to develop tools to determine the underlying mechanisms. Among these single-cell organisms, many bacterial species swim by using flagella consisting of a long extracellular filament, a hook, and a rotary bacterial flagellar motor anchored on the cell envelope (Figure 1) [1].

The flagellum consists of a thin helical flagellar filament that acts as a propeller, a reversible rotary molecular motor embedded on the envelope, and a hook that acts as a universal connection joint between the motor and the flagellar filament [2] (Figure 1). Flagellar distribution on the cell surface varies on different bacterial species. Peritrichous bacteria, such as *Escherichia coli* and *Salmonella enterica*, can produce multiple flagella distributed around the cell body. Monotrichous bacteria, such as *Vibrio alginolyticus*, have one single polar flagellum, and lophotrichous bacteria, such as *Vibrio fischeri*, have multiple flagella on one pole. The flagellar distribution affects bacterial swimming patterns as well as chemotaxis strategies. By switching motor rotation between counterclockwise to clockwise states, peritrichous *E. coli* can do a run-and-tumble swimming pattern [3]. Monotrichous *V. alginolyticus* can do forward-backward-turn through the flagellar flick [4,5].

The flagellar motor has a rotor and energy-conversion stator units that couple the ion-motive force and ion flux to the rotation. For example, *E. coli* and *Salmonella* use the proton (hydrogen nucleus), whereas marine *Vibrio* species use the sodium ion [6,7] (Figure 1). Recent reports have confirmed five MotA and two MotB proteins form one stator unit [8,9,10]. It is believed the ion flux passes through the stator unit ion channel coupled to the torque generation. Hence, a rotating flagellar motor can propel the cell body at a speed of 15–100 μm/s [11,12], and motor rotational speed is linear with proton motive force (PMF) [13]. The rotor comprises several stacked ring-link structures. The MS ring is composed of FliF, and the C ring is composed of FliG/FliM/FliN [14], which is located under the MS ring [15,16]. The rotary motor is driven by the interaction between the FliG and stator units to generate torque [17,18,19]. Moreover, a single stator unit can drive the rotor through conduction of at least 37 ions per revolution [20].

In this article, we reviewed the current understanding of bacterial flagellar filament constructions and the mechanisms of flagellar loss. A flagellar filament is typically about 5–20 μm (2–10 times the cell body length) and is a hollow cylinder with outer and inner diameters of 20 and 2 nm, respectively [21]. This long extracellular component is self-assembled with several thousand flagellin monomers [21]. The construction of flagellar filament is considered to occur in an inside-out manner, that is, flagellins are delivered from the base of the flagellar type-III secretion system (fT3SS), which attaches to the basal body of the flagellum [2,14,22]. Using energy derived from ATP hydrolysis and PMF [23,24,25], the fT3SS pumps unfolded flagellins into the flagellar channel. These unfolded flagellins are transported to the distal end and are then folded as the new part of the flagellar filament.

Although the structure and mechanical functions of bacterial flagella are well researched, the dynamics of flagellar loss remains poorly understood. Recent works have revealed that flagella are impermanent cellular structures, and cells can actively abandon this large motility apparatus [26,27,28,29,30]. Whereas molecular triggering and the activation mechanism remain unknown, the novel finding of the active ejection of the flagellar filament provides a new direction of cellular adaptation to external stimuli.

## 2. Bacterial Flagellin Transportation

### 2.1. Architecture of the Type-III Secretion System

The fT3SS is involved in the construction of the flagellar axial structure consisting of the rod, hook, and filament and the virulence-associated T3SS (vT3SS) of the injection device used by gram-negative bacteria injects toxic effectors into target cells [25]. The fT3SS and vT3SS have similar cytoplasmic components and an inner membrane export apparatus, but the final destinations of secreted proteins are different. The fT3SS keeps the secreted proteins to form the flagellar filaments while the vT3SS delivers the effectors into the target cells.

The basal body of the flagellar rotary motor is composed of an axial rod and MS, C, and LP rings (Figure 1). The MS ring consists of 33 subunits of protein FliF [31], and FliP/FliQ/FliR is assembled and embedded in the cytoplasm first during the flagellum formation on the membrane [32,33,34]. The C ring consists of 26–34 FliG [35], 34–44 FliM [36], and > 100 FliN [37] subunits and is located under the MS ring. All flagellar axial parts self-assemble through protein export of the fT3SS [38]. The core integral-membrane components of the flagellar export apparatus (FlhA/B and FliP/Q/R) are similar to the virulence-associated vT3SS [39]. Furthermore, fT3SS and vT3SS are powered by ATP hydrolysis and PMF [23,24,25,40,41,42]. Hence, during flagellar assembly, FliF first forms the MS ring in the cytoplasmic membrane. Next, FliG, FliM, and FliN assemble the C ring on the cytoplasmic side. FliG is also directly associated with the MS-ring component FliF [43,44]. After the basal body is complete, the axial proteins are exported through fT3SS [12,22,45,46].

**Figure 2:**
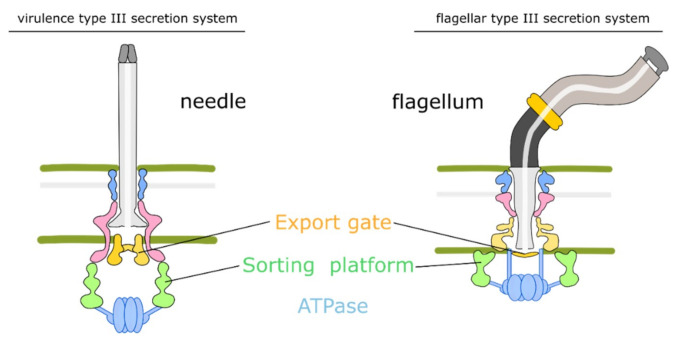
Schematic of vT3SS and fT3SS. The vT3SS and fT3SS are similar in terms of cytoplasmic components and the inner membrane export apparatus. ATPase associates with proteins to pump unfolded substrates into the export channel. Then, the sorting platform and export gate help line up the sequence of unfolded substrates. The flagellum consists of a rod, hook structure, and flagellar filament. The needle connects with rings to directly pass through the outer membrane.

The T3SS needle complex or flagellum is composed of rings for supporting a needle filament or flagellar filament, which extend from the inner membrane, through the periplasmic space and peptidoglycan layer, to the outer membrane (Figure 2). It serves as the central channel for translocating proteins. Several additional proteins combine with the basal body to form a functioning needle complex [47,48]. SctI assembles between membranes forming an inner rod as a needle adaptor, which can anchor the needle filament or the flagellum [49,50,51,52]. Then, extending from the inner rod to the extracellular environment is the needle filament or flagellar filament composed of protein SctF or FliC, respectively [53]. Additionally, there are several proteins surrounding the cytoplasmic side of the membrane-spanning rings, which form the sorting platform complex [54] similar to the flagellar C ring complex [55]. 

### 2.2. Strcture of the Flagellin

The bacterial flagellar filament is a 5–20 μm long, thin, and hollow helical propeller with an outer diameter of 20 nm and an inner diameter of 2 nm. It is packed with flagellins of two conformations, L and R types [56,57]. Folded flagellin FliC consists of four domains (D0, D1, D2, and D3) and is shaped like “Γ” with vertical and horizontal lengths of approximately 140 and 110 Å, respectively. In total, 11 flagellins are packed into one round of the filament tube in which the D0/D1 domain forms the double tubular structure as the inner core and the D2/D3 domain forms the outer structure [21]. Most of the subunit interactions in the outer part are through polar-to-polar interaction, and hydrophobic interactions are small. By contrast, the inner tube is mostly hydrophobic for high stability of the flagellar filament. The narrow channel diameter would reduce the diffusion rate of the unfolded flagellins during transportation in the flagellar tube. More details would be discussed in Section 3.2.

### 2.3. Substrates Accumulation and Delivery

The fT3SS is similar to the vT3SS in that both have five proteins that form the export apparatus and connect with the MS ring [25] (Figure 2). The fT3SS plays a crucial role in bacterial flagellar motor system as the rod, hook, and filament subunits are transported via the fT3SS, which includes an ATPase complex (FliI, FliJ, and FliH), an export substrate-chaperon docking platform (FlhAc), and a transmembrane export gate (FlhAB and FliPQR). The ATPase complex (FliI and FliJ) connects with the C ring (FliG, FliM, and FliN) through an interaction between FliH and FliN. There are two components of fT3SS to collect substrates nearby the export gate to increase the binding efficiency [58]. One is the associated ATPase complex (FliHIJ), which is composed of an ATPase (FliI), a regulator of ATPase (FliH), and a central stalk protein (FliJ) [59,60,61]. The other one is the C ring, which is located under the MS ring. The ATPase complex can deliver proteins to the export gate after the cytoplasmic FliH2FliI complex recruit the substrate-chaperone complexes [61,62]. The C ring provides binding sites for substrate-chaperone complexes and promotes the accumulation of substrates near the export gate [63].

The ATPase and the T3SS export gate play an important role in cargo transfer across the inner membrane. In early studies, ATP has been considered as the main energy source for T3SS. Recent research studies have shown that the PMF provides the primary energy source [23,24,40,41,64,65]. PMF is the sum of electrical and chemical potential across the membrane. Bacteria use PMF for several import cellular functions such as ATP synthesis, active membrane transport, and flagellar motility. The stator proteins MotA and MotB couples the ion flux driven by PMF to the motor rotation [66]. Further investigation revealed that the electrical components of PMF contributes as the main energy source to flagellar protein export [67]. The ATPase may have dual roles for shuttling substrates to the export gate and enhancing the efficiency of the export apparatus. However, the exact mechanism of energy conversion for the protein translocation by the export apparatus remains unknown. Once proteins are translocated across the inner membrane, the substrate proteins diffuse through the narrow channel until they arrive at their site of assembly. There is no evidence of active transportation involved in the proteins’ transportation inside the channel. 

For example, for filament subunit FliC, the chaperone-subunit complex (FliS–FliC) binds with the cytoplasmic ATPase complex before loading into the export channel. Then, the chaperone–subunit complex goes through the export gate [62,68,69,70]. Finally, the flagellar filament subunits are translocated across the cell membrane into the export channel and folded to become part of the filament at the distal end. In gram-negative bacteria, there are two additional rings, L and P rings, surrounding the axial rod rotation. These rings act as bearings for the axial rod. In other words, they anchor to the outer membrane and peptidoglycan layer, respectively. Overall, proteins are delivered through the fT3SS apparatus by using energy from ATP hydrolysis and PMF [23,24,25,40,41,42]. Filament subunit transportation is discussed in detail in Section 3.

## 3. Flagellar Filament Construction

**Figure 3:**
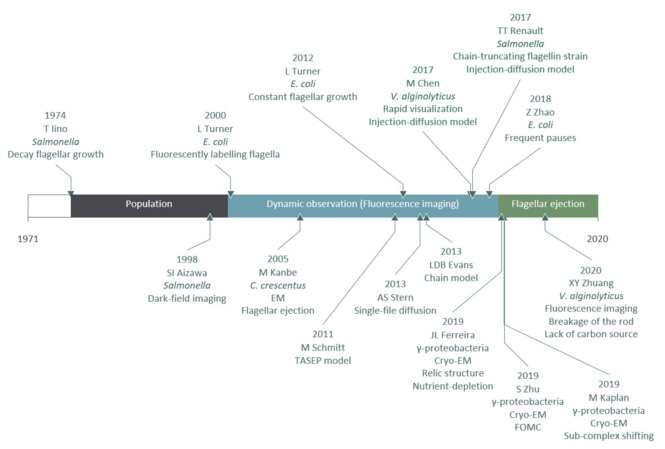
A timeline of experiments and models on flagellar filament construction and loss.

In this section, we outlined the crucial experimental and theoretical milestones in the study of the flagellar filament construction. A timeline of experiments and models on flagellar filament construction and loss is summarized in Figure 3.

### 3.1. Milestones of Flagellar Filament Growth Measurements

The first attempt to probe the flagellar filament growth rate can be dated back to 1974. At that time, the only tool for measuring flagellar filament length precisely was electron microscopy. Iino compared the flagellar length histogram at two different time points in a *Salmonella typhimurium* growing culture [71]. Assuming that the length order of two samples are the same, he calculated the growth rate of the flagellar filament from the filament length histograms. The most important finding from his experiments was that flagellar growth rate is length dependent with exponential decay formulated as follows.

(1) $$V=V_{0}e^{-KL}$$

where V is the flagellar length growth rate, V_0_ is the initial flagellar growth rate at L = 0, K is the decay rate, and L is the flagellar length. Iino found that the initial flagellar filament growth rate can be as high as 550 nm/min and that the fT3SS must secrete 18 FliC per second (Table 1).

In 1998, Aizawa and Kubori used dark-field microscopy to measure flagellar length distribution in different growth phases, and their results regarding flagellar growth rate were similar to those of Iino [72]. Both experiments show statistical filament growth from population data with limited temporal resolution. For measuring growth dynamics, development of flagellar filament fluorescent labelling is required [3].

In 2012, Turner et al. sequentially labeled *E. coli*’s flagellar filament with two colors of fluorophores and found that the average flagellar growth rate is independent of filament length [73]. The average filament growth rate was a constant (24 nm/min) but with high variation. These results motivate scientists in the field to revisit this long-standing question.

In 2017, based on improved in situ labeling and immunostaining, Renault et al. monitored the flagellar growth of *Salmonella* [74]. They provided a living-cell method for observing single cells dynamically growing individual flagella. Their results reported the length-dependent elongation mechanism with elongation speed decreasing gradually from approximately 100 (nm/min) to 20 (nm/min), thus, confirming the results of Iino [71].

Simultaneously, Chen et al. used the sheathed flagellar filament and fast easy sheath labeling of *V. alginolyticus* to largely improve temporal resolution of flagellar growth dynamics [75]. The sheath is a membrane-like structure and contiguous of the outer membrane [76,77,78]. The sheath can be easily labeled using lipophilic fluorescent dyes within a sub-second time scale [79,80]. Furthermore, the single polar flagellum system of *V. alginolyticus* reduces the difficulty faced during image analysis in separating entangled flagella. This report revealed that the flagellar growth process is highly length-dependent with an initial constant growth rate and then a decaying growth rate. The initial growth rate is approximately 50 nm/min (Table 1).

In 2018, Zhao et al. revisited *E. coli* flagellar growth by using biarsenical dyes to label flagellin with tetracysteine tag for single-cell observation [81] and showed that the flagellar growth of *E. coli* has frequent pauses due to insufficient flagellins [82]. Although a high fluctuation of the filament growth rate was observed similarly to that observed by Tuner. The average growth rate also decayed. 

With improved fluorescent labelling techniques, flagellar filament growth was observed in live single cells and showed a length-dependent decay in the growth rate. Temporal and spatial resolutions can be further improved to reveal the initial flagellar growth rate and transportation details.

### 3.2. Models for Flagellin Transport and Filament Growth

Once the secreted flagellin passes across the inner membrane, it continuously travels through the flagellar filament that could be up to 10 μm long. The filament central channel is too narrow for folded flagellin to pass. The energy source and transportation mechanism are the main mysteries.

The simplest model for flagellin transportation is that partially unfolded flagellins diffuse through the channel in a single-file and then fold at the distal end. Considering this model, Schmitt and Stark used the totally asymmetric simple exclusion process (TASEP) models with open boundary to simulate flagellin transportation and flagellar growth [83]. TASEP has been applied successfully to study nonequilibrium steady states such as the motion of ribosomes along mRNA, molecular motors along microtubule filaments, or the traffic of the car on the highway. In the TASEP model, particle diffusion is described based on the forward/backward rate with lattice sites. The single-file feature is simulated using particles that can only move when the target site is empty. This Monte Carlo simulation has four parameters, namely loading rate, crystallization rate, forward rate, and backward rate. With a small negative drift, that is, the forward rate smaller than the backward rate, the simulation data can match the experimental result of Iino. However, it was unclear why the filament channel exhibits biased Brownian diffusion of flagellin transportation.

In 2013, Stern and Berg performed a single-file diffusion simulation with more realistic parameters [84]. They assumed that the flagellin is unfolded into an α-helical chain, and the pump extrudes one flagellin every two seconds into a one-dimensional lattice tube. The diffusion process is modeled using the diffusion constant D. They found that, by changing the flagellin diffusion constant, the flagellar growth could be varied from a constant growth rate to a length-dependent decay rate. Furthermore, they used diffusion constants 30–480 times smaller than the estimated diffusion constant for an α-helical flagellin subunit diffused freely in water.

**Figure 4:**
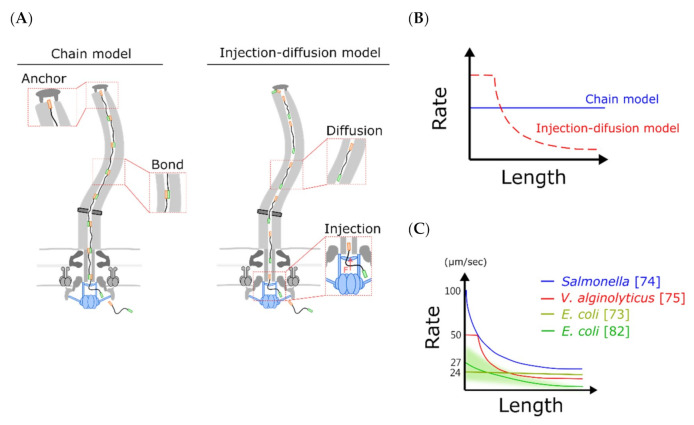
Potential mechanisms of the flagellar growth process. (**A**) Schematics of the chain model (left) and the injection-diffusion model (right) are shown. The chain model proposed that sequential flagellins are linked head-to-tail to form a chain, and the first flagellin anchors beneath the distal end of the flagellum to provide a pulling force. Therefore, constant force contributes to a constant growth rate. According to the injection-diffusion model, the secretion system applies a secretion force on an unfolded flagellin, and flagellins are delivered through diffusion after entering the channel. Hence, flagellins are crowded on the channel when the flagellum is getting longer. (**B**) The chain model predicts a constant growth rate, and the injection-diffusion model predicts a length-dependent growth rate. (**C**) The summary of flagellar growth rate of three bacteria using fluorescent-based techniques.

In 2017, Chen et al. built an injection-diffusion model to explain the high-resolution experimental data from *V. alginolyticus* flagella growth [75] with an initial constant growth rate and then a decayed growth rate (Figure 4A,C). The main difference of this model from the previous diffusion mechanism model is the addition of the pumping force at the secretion side that can push flagellin into the channel (Figure 4A). With Brownian dynamic simulation, they successfully reproduced the length-dependent growth rate and determined that the effective diffusion constant of flagellin in the channel is 1000 times smaller than that in the bulk water. With a reduction in the injection force, the initial constant growth rate region can be eliminated. Simultaneously, Renault et al. demonstrated the length-dependent *Salmonella* flagellar growth rate based on the injection-diffusion mechanism with analytical approaches [74].

To explain Tunner’s 2012 result of constant flagellar growth rate, Evans et al. proposed a chain mechanism model that harnesses the entropic force of the unfolded flagellins for the flagellar growth (Figure 4A,B) [85]. This model required all the transporting flagellins in the flagellar channel to form a long chain and the folding force of the distal end flagellin to pull the entire chain (Figure 4A,B). They demonstrated that a subunit docked at the export apparatus can be captured using a free subunit with a head-to-tail linkage of N-terminals and C-terminals. The pulling force adjusts as the flagellar length increases to maintain the constant flagellar growth rate. Although the chain model is physically simple, it is incompatible with some properties of flagellins [74]. First, N and C terminals of flagellin are anti-parallel in the linked chain but parallel in the folded flagellar structure. Second, the channel is too narrow to accommodate the linking regions of chains. Third, Renault et al. demonstrated that truncation of flagellin N-terminal and C-terminal linking region do not affect the flagellar growth rate [74]. Thus, it is less likely that the chain model is the fundamental mechanism of flagellin transportation.

The flagellin is delivered in a 2-nm narrow channel that is different from the bulk water environments. A decrease in an effective diffusion constant is expected. The decays of the flagellar growth rate are due to reduced transportation of long-distance diffusion and increased jamming (Figure 4C). However, the underlying reason for high variation in the measured initial flagellar growth rate is unclear (Table 1). A high flagellar growth rate of 550 nm/min requires transporting 18 flagellins per second. A new high-spatial-resolution real-time imaging method is required for further studies to reveal the mysterious high-speed initial growth rate and transportation mechanism.

## 4. Loss of Flagella

Bacterial flagellar motility is a fascinating feature of single-cell organisms. The construction of whole flagellum requires >20,000 proteins. Therefore, it is generally believed that constructing flagellum is costive and flagella are valuable for bacteria. However, a highly motile planktonic bacterial phase is only a part of the bacterial life cycle. Bacteria must switch between different phases and manage their flagellar motility. We have studied the construction sequence of the bacterial flagellar motor [2], but little is known regarding flagellar disassembly.

The unique life cycle of freshwater bacterium *Caulobacter crescentus* provides an opportunity to learn the potential mechanism of flagella loss through ejection. *C. crescentus* transitions between two distinguishable cell states known as a motile swarmer cell state with a polar flagellum and a surface-attached stalked cell state. In 2004, Grünenfelder et al. demonstrated that, in *C. crescentus*, ejection started from the inside out and reported that flagellar ejection is trigged by ClpA [86]. During differentiation into a stalked cell state, the swarmer cell releases its single polar flagellum. The flagellum released is synchronized with cell differentiation, and the ejected flagellum has a hook and partial rod of approximately 18 nm [30]. The breaking point of the structure can be localized to the MS ring–rod junction. Thus, two potential flagellum-releasing models are available for *C. crescentus*–destruction of the MS ring or breakage between the MS ring and the rod [30]. Moreover, the assembly and loss of the polar flagellum in symbiont *V. fischeri* [87] and plant-associated *Methylobacteria* [88] have been reported, but the mechanism remains unclear.

In the beginning, scientists focused only on the released flagellar structure. However, in 2019, multiple groups simultaneously noted another important finding. These groups observed a flagellar outer membrane complex (FOMC) [26,27,28] in different bacterial species by using cryo-electron tomography. A common feature of the FOMC is containing L and P rings of the bacterial flagellar motor with a plug located inside the P ring and without the hook, the flagellar filament, and the MS ring. This finding raises a new question of whether the FOMC is a precursor or a relic of bacterial flagella.

To further investigate the FOMC and its relation to the fully assembled flagellar motor, these groups also imaged different mutants disrupting the flagellar construction sequence. The FOMC could not be detected in strains lacking the rod protein, FlgG (e.g., *Pseudomonas aeruginosa*) [27,28], or intrinsic flagellar type III secretion system protein, FlhA (e.g., *Shewanella putrefaciens*) [26]. These results strongly suggest that the FOMC cannot form in the absence of the distal rod and secretion system. These results support previous models that suggest that P and L rings form around the assembled rod. Therefore, the FOMC is not a precursor of the flagellar motor assembly process.

Evidence suggests that bacteria can somehow disassemble their flagella and leave FOMC or a relic structure in the membrane. Since the flagellar motor is used for swimming, Ferreira et al. demonstrated that the swimming speed and the number density of flagellar motors of *Plesiomonas shigelloides* and *V. fischeri* decrease at high cell density in the growth medium [26]. Later, Zhuang et al. used fluorescent labeled *V. alginolyticus* single polar flagellum and measured the percentage of flagellated bacteria (PFB) during the *V. alginolyticus* growth. They found that the PFB increase rapidly in the early exponential phase through widespread flagella production. The PFB peaks at approximately 76% in the mid-exponential phase. After entering the stationary phase, the PFB begins to decline due to cessation of flagella production in daughter cells. When the cells enter the prolonged stationary phase, the flagellated cell concentration suddenly drops, indicating that the bacteria actively abandon flagella [29]. In their study, the swimming speed of *V. alginolyticus* was strongly correlated to the PFB, which is consistent with the finding of Ferreira et al. [26].

To catch the flagella ejection event, Ferreira et al. presented a striking image from in situ cryo-EM, showing a flagellar filament with a hook and short distal rod breaking off from the motor [26]. More importantly, Zhuang et al. recorded time-lapse images showing the polar flagellum of *V. alginolyticus* being ejected from the cell pole [29]. These single-cell experimental results confirmed that these bacterial cells actively eject their flagella. However, the trigger is unknown.

Ferreira et al. first demonstrated that the depletion of nutrients triggers ejection [26]. Further studies on *V. alginolyticus* showed that a lack of carbon source promotes flagellar disassembly. Whether flagella ejection has a universal trigger or has a species-dependent trigger is unknown. Further investigation is required to determine the molecular-level triggers.

**Figure 5:**
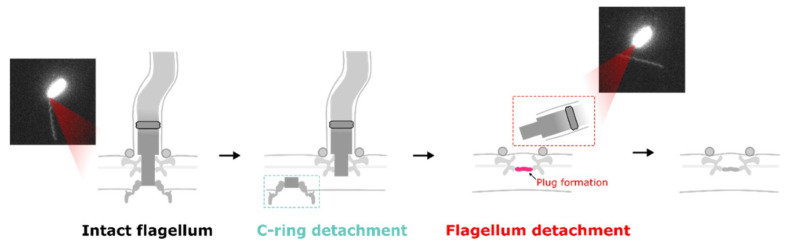
A model summarizing the disassembly process of a V. alginolyticus flagellum, showing that it begins with breaking the rod above the MS ring before FliG depolymerization. The C ring, with inner membrane components, then mobilizes on the cell membrane. Finally, the LP ring is likely sealed and the flagellum is ejected.

The mechanism of flagellar filament release by bacteria is not clear. In *C. crescentus*, the protease ClpAP is associated with FliF degradation. However, the depletion of ClpA and ClpX did not prevent flagellar ejection in *S. putrefaciens* [26]. Again, we can have a hint from in situ cryo-EM images. Kaplan et al. found inner-membrane complexes (C and MS rings) near FOMC (P and L rings). Zhuang et al., by using the single-molecular tracking of GFP-fused FliG of *V. alginolyticus*, found fast movement of FliG clusters on the inner membrane before flagellar filament loss. These results together suggest the “break the rod” model for flagellar filament release (Figure 5).

The FOMC are relics of ejected flagella but not flagellar assembly intermediates [26]. These findings raise the question of why bacteria do not keep the flagella as permanent cellular structures. A flagellum is composed of ~2 × 10^4^ proteins, which is a significant fraction of the total of 3 × 10^6^ proteins for a bacterial cell [29]. Building and rotating the flagella are energy-consuming for bacteria. However, several known mechanisms enable bacteria to stop flagellar rotation. For instance, with nutrient depletion, the activation of cyclic di-GMP signaling triggers YcgR, which is a c-di-GMP binding protein, to interact with the flagellar switch-complex proteins FliG and FliM, stopping flagellar rotation and acting as a “molecular brake” [89,90]. Additionally, reducing the ion motive force dissociates stator units from the flagellar motor in *E. coli* and *V. alginolyticus* [91]. The main purpose of active flagella loss under starvation is unclear. 

Although flagella play a crucial role in bacterial motility, flagellins are also essential antigens that can stimulate both innate inflammatory response and adaptive immunity development [92,93,94]. Two specialized receptors on immune cells, cell surface Toll-like receptor 5 (TLR5) [95,96,97], and intracellular receptor Ipaf [94,98,99] are responsible for recognizing flagellins as a warning of a pathogenic bacterial invasion. Hence, flagella ejection may be a common feature of flagellated bacteria. Further investigation is required on the active response of flagella ejection.

## 5. Concluding Remarks and Future Perspective

The self-assembly of tens of thousands of flagellins into an extracellular filament is an amazing process. Current data support the injection-diffusion mechanism for flagellin transportation and length-dependent decay in the flagellar growth rate. In the near future, investigating the efficiency of the secretion system, the initial flagellar growth rate, and the transportation mechanism will be noteworthy.

The active ejection of flagella causes flagellum loss and is different from the flagellar loss by shearing [100,101]. We speculate the presence of some trigger signals and a signal transduction pathway [102]. Moreover, a molecular level understanding is required to understand the “braking rod” process. Certainly, a significant amount of information needs to be explored regarding the flagellar motor, which is a phenomenal, tiny molecular machine.

## Figures and Tables

**Table 1:** Summary of flagellar filament growth in different species.

Species	V_0_ (nm/min)	Secretion Rate (#/sec)	Microscopy	Reference
*Salmonella*	550 (Decay)	18.33	Electron microscopy	[71]
	Decay		Dark-field imaging	[72]
	100 (Decay)	3.33	Fluorescence imaging	[74]
*E. coli*	24 (Constant)	0.80	Fluorescence imaging	[73]
	27 (Decay, pauses)	0.90	Fluorescence imaging	[82]
*V. alginolyticus*	50 (Decay)	1.67	Fluorescence imaging	[75]

## References

[1] Berg H.C., Anderson R.A. (1973). Bacteria swim by rotating their flagellar filaments. Nature.

[2] Macnab R.M. (2003). How bacteria assemble flagella. Annu. Rev. Microbiol..

[3] Turner L., Ryu W.S., Berg H.C. (2000). Real-time imaging of fluorescent flagellar filaments. J. Bacteriol..

[4] Seishi K., Imai N., Nishitoba M., Sugiyama S., Magariyama Y. (2005). Asymmetric swimming pattern of *Vibrio alginolyticus* cells with single polar flagella. FEMS Microbiol. Lett..

[5] Taute K.M., Gude S., Tans S.J., Shimizu T.S. (2015). High-throughput 3D tracking of bacteria on a standard phase contrast microscope. Nat. Commun..

[6] Manson M.D., Tedesco P., Berg H.C., Harold F.M., Van Der Drift C. (1977). A protonmotive force drives bacterial flagella. Proc. Natl. Acad. Sci. USA.

[7] Hirota N., Kitada M., Imae Y. (1981). Flagellar motors of alkalophilic bacillus are powered by an electrochemical potential gradient of Na^+^. FEBS Lett..

[8] Hennell-James R., Deme J., Alcock F., Silale A., Lauber F., Berks B.C., Lea S.M., Kjaer A. (2020). Structure of a proton-powered molecular motor that drives protein transport and gliding motility. bioRxiv.

[9] Deme J.C., Johnson S., Vickery O., Muellbauer A., Monkhouse H., Griffiths T., James R.H., Berks B.C., Coulton J.W., Stansfeld P.J. (2020). Structures of the stator complex that drives rotation of the bacterial flagellum. Nat. Microbiol..

[10] Santiveri M., Roa-Eguiara A., Kühne C., Wadhwa N., Berg H.C., Erhardt M., Taylor N.M.I. (2020). Structure and function of stator units of the bacterial flagellar motor. Cell.

[11] Furuno M., Atsumi T., Yamada T., Kojima S., Nishioka N., Kawagishi I., Homma M. (1997). Characterization of polar-flagellar-length mutants in *Vibrio alginolyticus*. Microbiology.

[12] Xue R., Ma Q., Baker M.A., Bai F. (2015). A delicate nanoscale motor made by nature—The bacterial flagellar motor. Adv. Sci..

[13] Gabel C.V., Berg H.C. (2003). The speed of the flagellar rotary motor of *Escherichia coli* varies linearly with protonmotive force. Proc. Natl. Acad. Sci. USA.

[14] Chevance F.F., Hughes K.T. (2008). Coordinating assembly of a bacterial macromolecular machine. Nat. Rev. Microbiol..

[15] Murphy G.E., Leadbetter J.R., Jensen G.J. (2006). In situ structure of the complete *Treponema primitia* flagellar motor. Nature.

[16] Thomas D.R., Francis N.R., Xu C., DeRosier D.J. (2006). The three-dimensional structure of the flagellar rotor from a clockwise-locked mutant of *Salmonella enterica serovar Typhimurium*. J. Bacteriol..

[17] Zhou J., Lloyd S.A., Blair D.F. (1998). Electrostatic interactions between rotor and stator in the bacterial flagellar motor. Proc. Natl. Acad. Sci. USA.

[18] Blair D.F. (2003). Flagellar movement driven by proton translocation. FEBS Lett..

[19] Takekawa N., Kojima S., Homma M. (2014). Contribution of many charged residues at the stator-rotor interface of the Na^+^-driven flagellar motor to torque generation in *Vibrio alginolyticus*. J. Bacteriol..

[20] Lo C.J., Sowa Y., Pilizota T., Berry R.M. (2013). Mechanism and kinetics of a sodium-driven bacterial flagellar motor. Proc. Natl. Acad. Sci. USA.

[21] Yonekura K., Maki-Yonekura S., Namba K. (2003). Complete atomic model of the bacterial flagellar filament by electron cryomicroscopy. Nature.

[22] Minamino T., Imada K., Namba K. (2008). Mechanisms of type III protein export for bacterial flagellar assembly. Mol. Biosyst..

[23] Minamino T., Namba K. (2008). Distinct roles of the FliI ATPase and proton motive force in bacterial flagellar protein export. Nature.

[24] Paul K., Erhardt M., Hirano T., Blair D.F., Hughes K.T. (2008). Energy source of flagellar type III secretion. Nature.

[25] Lee P.C., Rietsch A. (2015). Fueling type III secretion. Trends Microbiol..

[26] Ferreira J.L., Gao F.Z., Rossmann F.M., Nans A., Brenzinger S., Hosseini R., Wilson A., Briegel A., Thormann K.M., Rosenthal P.B. (2019). γ-proteobacteria eject their polar flagella under nutrient depletion, retaining flagellar motor relic structures. PLoS Biol..

[27] Kaplan M., Subramanian P., Ghosal D., Oikonomou C.M., Pirbadian S., Starwalt-Lee R., Mageswaran S.K., Ortega D.R., Gralnick J.A., El-Naggar M.Y. (2019). In situ imaging of the bacterial flagellar motor disassembly and assembly processes. EMBO J..

[28] Zhu S., Schniederberend M., Zhitnitsky D., Jain R., Galán J.E., Kazmierczak B.I., Liu J. (2019). In situ structures of polar and lateral flagella revealed by cryo-electron tomography. J. Bacteriol..

[29] Zhuang X., Guo S., Li Z., Zhao Z., Kojima S., Homma M., Wang P., Lo C., Bai F. (2020). Live-cell fluorescence imaging reveals dynamic production and loss of bacterial flagella. Mol. Microbiol..

[30] Kanbe M., Shibata S., Umino Y., Jenal U., Aizawa S.I. (2005). Protease susceptibility of the *Caulobacter crescentus* flagellar hook-basal body: A possible mechanism of flagellar ejection during cell differentiation. Microbiology.

[31] Johnson S., Fong Y.H., Deme J., Furlong E., Kuhlen L., Lea S.M. (2019). Structure of the bacterial flagellar rotor MS-ring: A minimum inventory/maximum diversity system. bioRxiv.

[32] Fabiani F.D., Renault T.T., Peters B., Dietsche T., Gálvez E.J.C., Guse A., Freier K., Charpentier E., Strowig T., Franz-Wachtel M. (2017). A flagellum-specific chaperone facilitates assembly of the core type III export apparatus of the bacterial flagellum. PLoS Biol..

[33] Fukumura T., Makino F., Dietsche T., Kinoshita M., Kato T., Wagner S., Namba K., Imada K., Minamino T. (2017). Assembly and stoichiometry of the core structure of the bacterial flagellar type III export gate complex. PLoS Biol..

[34] Kuhlen L., Abrusci P., Johnson S., Gault J., Deme J., Caesar J., Dietsche T., Mebrhatu M.T., Ganief T., Macek B. (2018). Structure of the core of the type III secretion system export apparatus. Nat. Struct. Mol. Biol..

[35] Suzuki H., Yonekura K., Namba K. (2004). Structure of the rotor of the bacterial flagellar motor revealed by electron cryomicroscopy and single-particle image analysis. J. Mol. Biol..

[36] Lele P.P., Branch R.W., Nathan V.S., Berg H.C. (2012). Mechanism for adaptive remodeling of the bacterial flagellar switch. Proc. Natl. Acad. Sci. USA.

[37] Zhao R., Pathak N., Jaffe H., Reese T.S., Khan S. (1996). FliN is a major structural protein of the C-ring in the *Salmonella typhimurium* flagellar basal body. J. Mol. Biol..

[38] Macnab R.M. (2004). Type III flagellar protein export and flagellar assembly. Biochim. Biophys. Acta.

[39] Cornelis G.R. (2006). The type III secretion injectisome. Nat. Rev. Microbiol..

[40] Galperin M.Y., Dibrov P.A., Glagolev A.N. (1982). ΔμH+ is required for flagellar growth in *Escherichia coli*. FEBS Lett..

[41] Wilharm G., Lehmann V., Neumayer W., Trcek J., Heesemann J. (2004). *Yersinia enterocolitica* type III secretion: Evidence for the ability to transport proteins that are folded prior to secretion. BMC Microbiol..

[42] Lee P.C., Stopford C.M., Svenson A.G., Rietsch A. (2010). Control of effector export by the *Pseudomonas aeruginosa* type III secretion proteins PcrG and PcrV. Mol. Microbiol..

[43] Ogawa R., Abe-Yoshizumi R., Kishi T., Homma M., Kojima S. (2015). Interaction of the C-terminal tail of FliF with FliG from the Na+-driven flagellar motor of *Vibrio alginolyticus*. J. Bacteriol..

[44] Lynch M.J., Levenson R., Kim E.A., Sircar R., Blair D.F., Dahlquist F.W., Crane B.R. (2017). Co-folding of a FliF-FliG split domain forms the basis of the MS:C ring interface within the bacterial flagellar motor. Structure.

[45] Erhardt M., Namba K., Hughes K.T. (2010). Bacterial nanomachines: The flagellum and type III injectisome. Cold Spring Harb. Perspect. Biol..

[46] Galan J.E., Lara-Tejero M., Marlovits T.C., Wagner S. (2014). Bacterial type III secretion systems: Specialized nanomachines for protein delivery into target cells. Annu. Rev. Microbiol..

[47] Miletic S., Goessweiner-Mohr N., Marlovits T.C., Wagner S., Galan J.E. (2020). The structure of the type III secretion system needle complex. Bacterial Type III Protein Secretion Systems.

[48] Minamino T., Kawamoto A., Kinoshita M., Namba K., Wagner S., Galan J.E. (2020). Molecular organization and assembly of the export apparatus of flagellar type III secretion systems. Bacterial Type III Protein Secretion Systems.

[49] Marlovits T.C., Kubori T., Sukhan A., Thomas D.R., Galán J.E., Unger V.M. (2004). Structural insights into the assembly of the type III secretion needle complex. Science.

[50] Marlovits T.C., Kubori T., Lara-Tejero M., Thomas D., Unger V.M., Galán J.E. (2006). Assembly of the inner rod determines needle length in the type III secretion injectisome. Nature.

[51] Hu J., Worrall L.J., Hong C., Vuckovic M., Atkinson C.E., Caveney N., Yu Z., Strynadka N.C.J. (2018). Cryo-EM analysis of the T3S injectisome reveals the structure of the needle and open secretin. Nat. Commun..

[52] Torres-Vargas C.E., Kronenberger T., Roos N., Dietsche T., Poso A., Wagner S. (2019). The inner rod of virulence-associated type III secretion systems constitutes a needle adapter of one helical turn that is deeply integrated into the system’s export apparatus. Mol. Microbiol..

[53] Kubori T., Sukhan A., Aizawa S.I., Galán J.E. (2000). Molecular characterization and assembly of the needle complex of the *Salmonella typhimurium* type III protein secretion system. Proc. Natl. Acad. Sci. USA.

[54] Lara-Tejero M., Kato J., Wagner S., Liu X., Galán J.E. (2011). A sorting platform determines the order of protein secretion in bacterial type III systems. Science.

[55] Francis N.R., Sosinsky G.E., Thomas D., DeRosier D.J. (1994). Isolation, characterization and structure of bacterial flagellar motors containing the switch complex. J. Mol. Biol..

[56] Kamiya R., Asakura S., Wakabayashi K., Namba K. (1979). Transition of bacterial flagella from helical to straight forms with different subunit arrangements. J. Mol. Biol..

[57] Yamashita l., Hasegawa K., Suzuki H., Vonderviszt F., Mimori-Kiyosue Y., Namba K. (1998). Structure and switching of bacterial flagellar filaments studied by X-ray fiber diffraction. Nat. Struct. Biol..

[58] Renault T.T., Guse A., Erhardt M., Wagner S., Galan J.E. (2020). Export mechanisms and energy transduction in type-III secretion machines. Bacterial Type III Protein Secretion Systems.

[59] Minamino T., Macnab R.M. (1999). Components of the *Salmonella flagellar* export apparatus and classification of export substrates. J. Bacteriol..

[60] Minamino T., MacNab R.M. (2000). FliH, a soluble component of the type III flagellar export apparatus of Salmonella, forms a complex with FliI and inhibits its ATPase activity. Mol. Microbiol..

[61] Ibuki T., Imada K., Minamino T., Kato T., Miyata T., Namba K. (2011). Common architecture of the flagellar type III protein export apparatus and F- and V-type ATPases. Nat. Struct. Mol. Biol..

[62] Bai F., Morimoto Y.V., Yoshimura S.D., Hara N., Kami-Ike N., Namba K., Minamino T. (2014). Assembly dynamics and the roles of FliI ATPase of the bacterial flagellar export apparatus. Sci. Rep..

[63] González-Pedrajo B., Minamino T., Kihara M., Namba K. (2006). Interactions between C ring proteins and export apparatus components: A possible mechanism for facilitating type III protein export. Mol. Microbiol..

[64] Erhardt M., Mertens M.E., Fabiani F.D., Hughes K.T. (2014). ATPase-independent type-III protein secretion in *Salmonella enterica*. PLoS Genet..

[65] Lee P.C., Zmina S.E., Stopford C.M., Toska J., Rietsch A. (2014). Control of type III secretion activity and substrate specificity by the cytoplasmic regulator PcrG. Proc. Natl. Acad. Sci. USA.

[66] Blair D.F., Berg H.C. (1990). The MotA protein of E. coli is a proton-conducting component of the flagellar motor. Cell.

[67] Minamino T., Morimoto Y.V., Hara N., Namba K. (2011). An energy transduction mechanism used in bacterial flagellar type III protein export. Nat. Commun..

[68] Bange G., Kümmerer N., Engel C., Bozkurt G., Wild K., Sinning I. (2010). FlhA provides the adaptor for coordinated delivery of late flagella building blocks to the type III secretion system. Proc. Natl. Acad. Sci. USA.

[69] Kinoshita M., Hara N., Imada K., Namba K., Minamino T. (2013). Interactions of bacterial flagellar chaperone–substrate complexes with FlhA contribute to co-ordinating assembly of the flagellar filament. Mol. Microbiol..

[70] Abrusci P., Vergara-Irigaray M., Johnson S., Beeby M.D., Hendrixson D.R., Roversi P., Friede M.E., Deane J.E., Jensen G.J., Tang C.M. (2013). Architecture of the major component of the type III secretion system export apparatus. Nat. Struct. Mol. Biol..

[71] Iino T. (1974). Assembly of Salmonella flagellin in vitro and in vivo. J. Supramol. Struct..

[72] Aizawa S.I., Kubori T. (1998). Bacterial flagellation and cell division. Genes Cells.

[73] Turner L., Stern A.S., Berg H.C. (2012). Growth of flagellar filaments of *Escherichia coli* is independent of filament length. J. Bacteriol..

[74] Renault T.T., Abraham A.O., Bergmiller T., Paradis G., Rainville S., Charpentier E., Guet C.C., Tu Y., Namba K., Keener J.P. (2017). Bacterial flagella grow through an injection-diffusion mechanism. eLife.

[75] Chen M., Zhao Z., Yang J., Peng K., Baker M.A.B., Bai F., Lo C.J. (2017). Length-dependent flagellar growth of *Vibrio alginolyticus* revealed by real time fluorescent imaging. eLife.

[76] Glauert A.M., Kerridge D., Horne R.W. (1963). The fine structure and mode of attachment of the sheathed flagellum of *Vibrio metchnikovii*. J. Cell. Biol..

[77] Allen R.D., Baumann P. (1971). Structure and arrangement of flagella in species of the genus Beneckea and *Photobacterium fischeri*. J. Bacteriol..

[78] McCarter L.L. (2001). Polar flagellar motility of the Vibrionaceae. Microbiol. Mol. Biol. Rev..

[79] Grossart H.P., Steward G.F., Martinez J., Azam F. (2000). A simple, rapid method for demonstrating bacterial flagella. Appl. Environ. Microbiol..

[80] Wu Y., Yeh F.L., Mao F., Chapman E.R. (2009). Biophysical characterization of styryl dye-membrane interactions. Biophys. J..

[81] Copeland M.F., Flickinger S.T., Tuson H.H., Weibel D.B. (2010). Studying the dynamics of flagella in multicellular communities of *Escherichia coli* by using biarsenical dyes. Appl. Environ. Microbiol..

[82] Zhao Z., Zhao Y., Zhuang X.Y., Lo W.C., Baker M.A.B., Lo C.J., Bai F. (2018). Frequent pauses in *Escherichia coli* flagella elongation revealed by single cell real-time fluorescence imaging. Nat. Commun..

[83] Schmitt M., Stark H. (2011). Modelling bacterial flagellar growth. Europhys. Lett..

[84] Stern A.S., Berg H.C. (2013). Single-file diffusion of flagellin in flagellar filaments. Biophys. J..

[85] Evans L.D.B., Poulter S., Terentjev E.M., Hughes C., Fraser G.M. (2013). A chain mechanism for flagellum growth. Nature.

[86] Grunenfelder B., Tawfilis S., Gehrig S., Østerås M., Eglin D., Jenal U. (2004). Identification of the protease and the turnover signal responsible for cell cycle-dependent degradation of the *Caulobacter* FliF motor protein. J. Bacteriol..

[87] Ruby E.G., Asato L.M. (1993). Growth and flagellation of *Vibrio fischeri* during initiation of the sepiolid squid light organ symbiosis. Arch. Microbiol..

[88] Doerges L., Kutschera U. (2014). Assembly and loss of the polar flagellum in plant-associated methylobacteria. Naturwissenschaften.

[89] Boehm A., Kaiser M., Li H., Spangler C., Kasper C.A., Ackermann M., Kaever V., Sourjik V., Roth V., Jenal U. (2010). Second messenger-mediated adjustment of bacterial swimming velocity. Cell.

[90] Paul K., Nieto V., Carlquist W.C., Blair D.F., Harshey R.M. (2010). The c-di-GMP binding protein YcgR controls flagellar motor direction and speed to affect chemotaxis by a “backstop brake” mechanism. Mol. Cell..

[91] Fukuoka H., Wada T., Kojima S., Ishijima A., Homma M. (2009). Sodium-dependent dynamic assembly of membrane complexes in sodium-driven flagellar motors. Mol. Microbiol..

[92] Honko A.N., Mizel S.B. (2005). Effects of flagellin on innate and adaptive immunity. Immunol. Res..

[93] Salazar-Gonzalez R.M., McSorley S.J. (2005). *Salmonella flagellin*, a microbial target of the innate and adaptive immune system. Immunol. Lett..

[94] Miao E.A., Andersen-Nissen E., Warren S.E., Aderem A. (2007). TLR5 and Ipaf: Dual sensors of bacterial flagellin in the innate immune system. Semin. Immunopathol..

[95] Hayashi F., Smith K.D., Ozinsky A., Hawn T.R., Yi E.C., Goodlett D.R., Eng J.K., Akira S., Underhill D.M., Aderem A. (2001). The innate immune response to bacterial flagellin is mediated by Toll-like receptor 5. Nature.

[96] Smith K.D., Ozinsky A. (2002). Toll-like receptor-5 and the innate immune response to bacterial flagellin. Curr. Top. Microbiol. Immunol..

[97] Smith K.D., Andersen-Nissen E., Hayashi F., Strobe K., Bergman M.A., Barrett S.L., Cookson B.T., Aderem A. (2003). Toll-like receptor 5 recognizes a conserved site on flagellin required for protofilament formation and bacterial motility. Nat. Immunol..

[98] Franchi L., Amer A., Body-Malapel M., Kanneganti T.D., Ozoren N., Jagirdar R., Inohara N., Vandenabeele P., Bertin J., Coyle A. (2006). Cytosolic flagellin requires Ipaf for activation of caspase-1 and interleukin 1beta in salmonella-infected macrophages. Nat. Immunol..

[99] Miao E.A., Alpuche-Aranda C.M., Dors M., Clark A.E., Bader M.W., Miller S.I., Aderem A. (2006). Cytoplasmic flagellin activates caspase-1 and secretion of interleukin 1β via Ipaf. Nat. Immunol..

[100] Vogler A.P., Homma M., Irikura V.M., Macnab R.M. (1991). *Salmonella typhimurium* mutants defective in flagellar filament regrowth and sequence similarity of FliI to F0F1, vacuolar, and archaebacterial ATPase subunits. J. Bacteriol..

[101] Rosu V., Hughes K.T. (2006). Sigma28-dependent transcription in *Salmonella enterica* is independent of flagellar shearing. J. Bacteriol..

[102] Zhu S., Gao B. (2020). Bacterial flagella loss under starvation. Trends Microbiol..

